# Fibrinogen and tumors

**DOI:** 10.3389/fonc.2024.1393599

**Published:** 2024-05-08

**Authors:** Xinyuan Wu, Xiaomin Yu, Cheng Chen, Chenlu Chen, Yuxin Wang, Dongyan Su, Liqing Zhu

**Affiliations:** ^1^ School & Hospital of Stomatology, Wenzhou Medical University, Wenzhou, Zhejiang, China; ^2^ Department of Clinical Laboratory, The First Affiliated Hospital of Wenzhou Medical University, Wenzhou, Zhejiang, China; ^3^ Department of Hematology, Wenzhou Key Laboratory of Hematology, The First Affiliated Hospital of Wenzhou Medical University, Wenzhou, Zhejiang, China; ^4^ Department of Clinical Laboratory, Peking University Cancer Hospital and Institute, Beijing, China

**Keywords:** fibrinogen, tumor, pro-inflammatory, angiogenesis, metastasis, molecular pathway

## Abstract

Elevated plasma fibrinogen (Fg) levels consistently correlate with an unfavorable prognosis in various tumor patient cohorts. Within the tumor microenvironment, aberrant deposition and expression of Fg have been consistently observed, interacting with multiple cellular receptors and thereby accentuating its role as a regulator of inflammatory processes. Specifically, Fg serves to stimulate and recruit immune cells and pro-inflammatory cytokines, thereby contributing to the promotion of tumor progression. Additionally, Fg and its fragments exhibit dichotomous effects on tumor angiogenesis. Notably, Fg also facilitates tumor migration through both platelet-dependent and platelet-independent mechanisms. Recent studies have illuminated several tumor-related signaling pathways influenced by Fg. This review provides a comprehensive summary of the intricate involvement of Fg in tumor biology, elucidating its multifaceted role and the underlying mechanisms.

## 1 Introduction

Fibrinogen (Fg), a 340 kDa dimeric glycoprotein, is synthesized by hepatocytes and composed of three polypeptide chains: fibrinogen Aα (FGA, 52.0 kDa), Bβ (FGB, 52.0 kDa), and γ (FGG, 46.5 kDa (1). Each Fg molecule comprises two outer D domains connected by a central E domain, with the E-region encompassing the N-terminus of Aα, Bβ, and γ chains, while the D-region consists of the C-terminus of the Bβ and γ chains (2).

Traditionally recognized as a coagulation factor, Fg undergoes transformation into a fibrin polymer during the clotting cascade. In its role as an acute-phase reaction protein, Fg actively engages in inflammatory processes and interacts with the activation and migration of leukocytes (3). Noteworthy, previous research has consistently reported elevated plasma Fg levels across various tumor types, underscoring its significant involvement in tumorigenesis (4).

Moreover, Fg deposition has been observed in the tumor extracellular matrix (5) and the walls of angiogenic blood vessels (6), implying associations between Fg and tumor angiogenesis and metastasis. These interactions are mediated by binding sites on Fg, which engage with diverse molecules, including various plasma proteins and cellular receptors (7). Furthermore, multiple pathways have been identified linking Fg to tumor progression (8). This comprehensive review provides an in-depth overview of the pivotal role played by Fg in tumor biology, emphasizing its crucial contributions to angiogenesis, metastasis, and inflammation within the tumor microenvironment.

## 2 The role and mechanism of Fg in tumors

### 2.1 Plasma Fg levels in tumor patient cohorts

Elevated plasma fibrinogen(Fg) levels consistently manifest in diverse tumor patient cohorts (9). In lung, rectal, and stomach cancers, heightened Fg levels correlate with poorer survival (10–12) are recognized as an independent prognostic marker (4). In lymphoma and leukemia, multivariate analysis underscores plasma Fg as a biomarker and a robust predictor.

**Table 1** furnishes a comprehensive overview delineating the multifaceted roles and associations of pre-treatment plasma Fg levels within diverse medical contexts. The presented findings underscore the versatile nature of Fg, positioning it as both a prognostic marker and a predictor across varying conditions, including considerations of tumor-node-metastasis (TNM) stage and responses to surgery or chemoradiotherapy.

**Table 1:** Application of Fg as prognostic indicators and biomarkers in tumors.

Tumor Cohort	Authors	Patients	Major findings	OS, overall survival		Odd ratio		DFS, disease-free survival		Recurrence-free survival	
				hazard ratio (95%CI)	P- value	Odd ratio (95%CI)	P- value	hazard ratio (95%CI)	P- value	hazard ratio (95%CI)	P- value
Respiratory tumors											
NPC	He et al (13)	998 patients from China	The pre-treatment plasma Fg level is an independent prognostic marker with the capacity to predict survival outcomes.	1.970 (1.324–2.931)	0.001	–	–	–	–	–	–
LSCC	Cai et al (12)	203 patients following therapy from China	Fg emerges as a pivotal marker in predicting survival outcomes.	1.992 (1.098-3.614)	0.023	–	–	1.658 (1.034-2.657)	0.036	–	–
SCLC	Fan et al. (14)	120 patients from China	Elevated plasma Fg serves as a reliable prognostic indicator.	1.505 (1.018–2.226)	0.041	–	–	–	–	–	–
NSCLC	Mitsui et al (15)	149 stage I NSCLC patients following lobectomy from Japan	Preoperative plasma Fg levels prove to be valuable predictors of both recurrence and survival.	5.147 (1.44-18.40)	0.012	–	–	–	–	3.076 (1.19-7.95)	0.02
	Sinn et al (16)	84 stage III/N2 NSCLC patients following neoadjuvant therapy from Austria	Decreased Fg predicts superior overall survival (OS).	0.994 (0.989,0.999)	0.025	–	–	0.996 (0.991-1.001)	0.136	–	–
Gastroenteric carcinoma											
Oral cancer	Su et al. (17)	116 patients following oral cancer surgery from China	Plasma Fg demonstrates diagnostic utility for identifying osteomyelitis of the jaws after oral cancer surgery.	–	–	–	–	–	–	–	–
OSCC	Wu et al (18)	365 patients following radical resection from China	Elevated plasma Fg is associated with a less favorable prognosis.	5.301 (2.426-11.581)	<0.001^*^	–	–	–	–	–	–
Esophageal Cancer	Wakatsuki et al. (19)	100 patients from following radical esophagectomy with two- or three-field lymphadenectomy Japan	Preoperative plasma Fg stands out as a potential biomarker for predicting tumor progression, recurrence patterns, and prognosis.	1.88 (1.06-3.29)	0.031^**^	–	–	–	–	–	–
	Hoshino et al (20)	438 esophageal squamous cell carcinoma (ESCC) patients following transthoracic esophagectomy from Japan	Fg serves as an important prognostic factor in ESCC.	2.36(1.66,3.35)	<0.001	–	–	–	–	2.27(1.65,3.14)	<0.001
GC	Cheng et al. (10)	8315 patients	Elevated plasma Fg serves as a potential predictor for worse OS and recurrence-free survival (RFS), correlating significantly with aggressive clinical features.	1.57 (1.36,1.81)	< 0.001	–	–	–	–	2.54 (1.19, 5.41)	0.016
	Wang et al (21)	542 advanced gastric cancer patients with Borrmann type III following radical gastrectomy from China	Patients with high Fg levels experience worse RFS and OS.	1.140 (1.058,1.228)	0.001	–	–	–	–	1.140 (1.059,1.228)	0.001
Colon cancer	Parisi et al (22)	126 patients from Italy	Elevated Fg levels indicate a higher risk and are linked to poorer OS.	1.91 (1.15, 3.17)	0.012^*^	–	–	–	–	–	–
Rectal cancer	Lee et al. (11)	947 patients receiving preoperative chemoradiotherapy and following radical surgery from Korea	Elevated Fg levels remain predictive after preoperative chemoradiotherapy.	–	–	2.026 (1.369,2.997)	<0.001	–	–	–	–
HCC	Huang et al (23)	1,961 patients from Asia	Elevated plasma Fg levels might predict poor prognosis and advanced tumor progression.	2.08 (1.67, 2.59)	< 0.0001	–	–	1.90 (1.52, 2.37)	< 0.0001	1.90 (1.52, 2.37)	< 0.0001
	Xu et al (24)	461 HCC patients following curative hepatectomy.	High Fg levels are associated HCC survival.	1.362 (1.183,1.567)	<0.001	–	–	–	–	–	–
PC	Guo et al (25)	133 patients from China	Elevated Fg levels could serve as predictors for distant metastasis.	–	–	5.666 (1.802,17.813)	0.003	–	–	–	–
	Chung et al (26)	67 pancreatic ductal adenocarcinoma patients from Korea	Serum Fg levels may predict prognosis.	1.906 (1.124,3.231)	0.017	–	–	–	–	–	–
HC	Ye et al. (27)	171 patients following curative-intent resection from China	High plasma Fg levels, independent of tumor stage, surgical margin, vascular invasion, and lymph-node metastasis, are associated with poor outcomes.	1.541 (1.044,2.274)	0.029	–	–	–	–	–	–
GBC	Cao et al. (28)	58 patients following surgery from China	Plasma Fg levels act as prognostic factors predicting outcomes following surgery.	1.012 (0.682,1.876)	<0.001	–	–	–	–	–	–
GIST	Lu et al. (29)	91 patients following curative-intent resection from China	Elevated plasma Fg stands out as an independent prognostic biomarker.	3.90 (1.90,8.15)	<0.001	–	–	–	–	2.43 (1.40,4.06)	0.002
Hematological malignancy											
DLBCL	Troppan et al. (30)	372 patients from Austria	High plasma Fg levels at diagnosis predict poor outcomes.	1.69 (1.06,2.72)	0.029	–	–	–	–	1.68 (1.08,2.61)	0.021
AML	Zhang et al. (31)	2947 patients	Plasma Fg levels are related to OS.	1.21 (1.01,1.44)	<0.001	–	–	–	–	–	–
LBCL	Holtzman et al. (32)	45 patients with relapsed/refractory LBCL treated with axicabtagene ciloleucel from US	Elevated Fg levels at baseline could predict the risk of immune effector cell-associated neurotoxicity syndrome.	–	–	–	–	–	–	–	–
Urogenital Neoplasms											
RCC	Tian et al (33)	3744 patients	Elevated plasma Fg levels indicate poor prognosis.	2.13 (1.74,2.61)	<0.001	–	–	1.67 (1.30, 2.15)	<0.001	–	–
UTUC	Liu et al. (34)	130 non-metastatic UTUC patients following surgery from China	Increased preoperative plasma Fg is an independent prognostic factor	–	–	–	–	–	–	–	–
Cervical cancer	Polterauer et al. (35)	313 patients following conization or simple hysterectomy from Austria	Plasma Fg is an independent prognostic parameter.	1.7 (1.3,2.1)	<0.001	–	–	1.7 (1.4, 2.1)	<0.001	–	–
Ovarian cancer	Seebacher et al. (36)	241 patients with adnexal masses following surgery from Austria	Plasma Fg is a robust predictor.	–	–	3.52 (2.26, 5.48)	<0.001	–	–	–	–
	Farzaneh et al (37)	141 patients from Iran	Plasma Fg levels can independently predict malignant ovarian tumors.	–	–	1.012 (0.998, 1.025)	0.07	–	–	–	–
	Hefler-Frischmuth (38)	224 patients from Austria	Elevated Fg levels are independently related to malignant ovarian tumors.	–	–	2.4 (1.4,4.0)	0.002	–	–	–	–
Uterine leiomyosarcoma	Bekos et al. (39)	70 patients from Austria	High plasma Fg levels are linked to aggressive tumor biology and an unfavorable prognosis.	1.3 (0.60,2.83)	0.51	–	–	–	–	–	–
Endometrial cancer	Seebacher et al (40)	436 patients from Austria	Plasma Fg is an independent prognostic parameter.	1.4 (1.1,1.2)	0.01	–	–	1.3 (1.01,1.6)	0.04	–	–
Prostatic cancer	Wang et al (41)	290 patients following deprivation therapy from China	Pretreatment plasma Fg level are related to tumor progression and the prognosis.	1.965 (1.181,3.270)	0.009	–	–	–	–	–	–
Bladder tumor	Li et al (42)	206 non-muscle-invasive bladder cancer from following transurethral resection China	Preoperative Plasma Fg is a prognostic biomarker.	–	–	–	–	–	–	1.593 (1.049,2.421)	0.029
Other tumors											
Breast cancer	Graf et al. (43)	114 nulliparous patients from Germany	Elevated Fg levels predict the risk of breast cancer [HR=6.53, 95%CI:1.76-24.3, P=0.01].	–	–	–	–	–	–	–	–
	Wang et al (44)	1004 patients with invasive breast cancer following neoadjuvant chemotherapy and subsequent surgery from China	Increased Fg levels predict a worse prognosis.	–	–	3.038 (1.667,5.537)	<0.001	–	–	–	0.951
PTC	Liu et al. (45)	1023 PTC patients following surgery from China	Patients with hyperfibrinogenemia were possible to an advanced TNM stage and a higher recurrence rate.	–	–	2.891 (1.201,4.874)	0.032	–	–	4.228 (2.102,7.541)	0.007
GBMs	Wang et al. (46)	315 patients following surgery from China	Elevated plasma Fg predicts a shorter OS outcome.	0.64 (0.41,1.00)	0.048	–	–	–	–	–	–
MPM	Ghanim et al. (47)	176 patients receiving curative resection, chemo- and/or radiotherapy from Austria	Fg is identified as an independent prognostic biomarker.	1.81 (1.23,2.65)	<0.01	–	–	–	–	–	–
Soft tissue tumor	Asanuma et al. (48)	102 patients from Japan	Elevated Fg levels are recognized as an important predictor.	–	–	6.452 (2.320,17.857)	0.0004	–	–	–	–
Osteosarcoma	Pu et al. (49)	160 patients following surgery from China	Higher Fg levels are associated with a potential for worse OS and PFS.	1.069 (0.625,1.826)	0.808	–	–	1.145 (0.667,1.965)	0.624	–	–
Liposarcoma	Peschek et al. (50)	158 patients following surgery from Austria	Elevated Fg are linked to adverse OS.	1.04 (1.02,1.06)	< 0.001	–	–	–	–	–	–

OS, overall survival; RFS, recurrence-free survival; NPC, nasopharyngeal carcinoma; LSCC, laryngeal squamous cell carcinoma; SCLC, small cell lung cancer; NSCLC, non-small cell lung cancer; OSCC, oral squamous cell carcinoma; GC, gastric cancer; HCC, hepatocellular carcinoma; GBC, Gallbladder cancer; PC, pancreatic cancer; HC, hilar cholangiocarcinoma; GIST, gastrointestinal stromal tumor; DLBCL, diffuse large B cell lymphoma; AML, acute myeloid leukemia; LBCL, large B-cell lymphoma; RCC, renal cell carcinoma; UTUC, upper urinary tract urothelial carcinoma; PTC, papillary thyroid carcinoma; GBMs, glioblastomas; MPM, malignant pleural mesothelioma;

^*^multivariate analyses of prognostic factors in patients.

^**^multivariate analyses of prognostic factors for overall survival and recurrence-free survival in patients.

Notably, although the prognosis of Fg has been demonstrated in most tumors, the prognosis of Fg in certain tumors remains controversial. In patients with relapsed and/or metastatic head and neck squamous cell carcinoma (R/M HNSCC), Fg levels may serve as a predictive indicator for survival (51). However, insights from Nenclares’ research on oral squamous cell carcinoma (OSCC) patients suggest that Fg levels lack predictive value in this context (52). Additionally, it is noteworthy that decreased plasma Fg levels are indicative of poor prognosis in angiosarcoma of the head and neck (ASHN) (53) and acute promyelocytic leukemia (APL) (54), as reported in the literature.

Elevated Fg levels in patients undergoing treatment for various tumors often correlate with poor prognosis, offering clinicians valuable insights for treatment planning and patient management. In surgical settings, heightened preoperative Fg levels typically signal a bleak prognosis post-surgery, warranting vigilant monitoring. Regarding chemotherapy, patients with hepatocellular carcinoma and elevated preoperative plasma Fg levels tend to exhibit poor responses to transarterial chemoembolization (TACE) (55). Similarly, in rectal cancer patients undergoing chemoradiotherapy (CRT) with radiotherapy, those with lower pre-treatment Fg levels (≤270 mg/mL) are more than twice as likely to achieve complete remission compared to counterparts with higher pre-treatment Fg levels (>270 mg/mL) (11). Moreover, in esophageal squamous cell carcinoma (ESCC), patients with elevated Fg levels demonstrate reduced responsiveness to navumab (20). These findings underscore the importance of tailoring chemotherapy regimens to individual cancer patients with elevated Fg levels to optimize treatment efficacy. Therefore, prioritizing the assessment of elevated Fg levels before treatment is imperative, enabling clinicians to customize treatment strategies and accurately evaluate post-treatment prognosis for cancer patients.

### 2.2 Fg in tumor extracellular matrix

Research has unveiled the presence of Fg in the tumor stroma, predominantly composed of connective tissues, inflammatory cells, and newly formed blood vessels (56). Elevated Fg expression has been detected in various original tumor biopsy samples, encompassing patients with breast cancer (57), uterine cervix carcinoma (58) and HCC (5), among others. Mechanistic insights into Fg expression in the tumor mesenchyme vary across different tumor settings. In cases of central nervous system lymphoma, disruption of the blood-brain barrier contributes to enhanced Fg deposition within the tumor stroma (59). In breast cancer, the binding interactions between Fg β15-42 and VE-cadherin improve endothelial barrier permeability (60). Increased vascular permeability within tumors results in the leakage of Fg from plasma into the tumor stroma (61). Notably, Fg deposition in acute promyelocytic leukemia is mediated by Fg binding to CD44 on APL blasts and NB4 cells (62).

Furthermore, tumor cells, including lung adenocarcinoma A549 cells (63), hepatocellular carcinoma HepG2 cells (64), human breast carcinoma MCF-7 cells (57) and uterine cervix carcinoma ME-180 cell (58), have been identified as capable of synthesizing and secreting Fg. This synthesis is attributed to elevated transcription of fibrinogen Aα (FGA), fibrinogen Bβ (FGB), and fibrinogen γ (FGG) genes.

**Table 2** provides a comprehensive summary of diverse observations related to Fg expression across different pathological contexts.

**Table 2:** Fg in ECMs and tumor cell lines.

Cancer Cohort	Methods	Major finding	References
NSCLC	Immunohistochemical	Fibrinogen γ (FGG) levels in tissues are observed within the nuclei.	(65)
GC	WB and RT-PCR Immunohistochemical	Gastric cancer tissues express fibrinogen Aα (FGA).	(66)
Colorectal adenocarcinoma	Immunofluorescence and immunohistochemical	Colorectal adenocarcinoma biopsies demonstrate elevated Fg deposition, bound to the tumor surface and adjacent to overlying dermal tissue.	(67, 68)
HCC	WB, RT-PCR and immunohistochemical	HCC tissue exhibits a significant and dramatic increase in Fg expression.	(5, 69)
PC	Immunohistochemical	Pancreatic cancer stromal tissues are examined for Fg expression.	(70)
CNS B-Cell Lymphoma	Immunohistochemical and immunofluorescence	Abundant Fg deposition characterizes specimens of CNS B-cell lymphoma.	(59)
RCC	Western blot, RT-PCR and immunohistochemical	Elevated extravascular Fg expression is identified adjacent to tumor cells or around blood vessels.	(71, 72)
Endometrial cancer	Immunohistochemical	Elevated Fg expression is observed in specimens of endometrial cancer.	(73)
Breast cancer	Immunostaining, RT-PCR and southern hybridization	Molecular analyses, including Southern hybridization, demonstrate the presence of fibrinogen Aα (FGA), fibrinogen Bβ (FGB), and fibrinogen γ (FGG) chain genes in MCF-7 cells. Immunostaining further reveals extracellular Fg adjacency to the surface of MCF-7 cells.	(57)
GBMs	Immunofluorescence	Fg expression is notably higher in tumor specimens.	(74)
FLC	Immunohistochemical	In FLC of the liver, Fg levels Fg levels surpass those in HCC.	(75)

NSCLC, non-small cell lung cancer; GC, gastric cancer; HCC, hepatocellular carcinoma; PC, pancreatic cancer; CNS B-Cell Lymphoma, Central Nervous System B-Cell Lymphoma; RCC, renal cell carcinoma; GBMs, glioblastomas; FLC, fibrolamellar carcinoma.

### 2.3 Pro-inflammatory role of Fg in tumors

Inflammation serves as a driving force across all stages of carcinogenesis, fostering cancer growth. The creation of an inflammatory tumor microenvironment (TME) results from intricately coordinated interactions involving cancer cells, stromal cells, and inflammatory cells (76). Additionally, tumor cells actively contribute to crucial stages of leukocyte recruitment and trafficking (77). These processes encompass chemokine-induced migration, coupled with the activation of leukocyte integrins and selectin adhesion molecules, facilitating the interaction of tumor cells with vascular endothelium and subsequent extravasation.

Vascular and leukocyte responses play pivotal roles in the pathogenesis of inflammation. Elevated vascular permeability is among the initial responses to inflammation. Fibrinogen (Fg) plays a role in facilitating the subsequent transendothelial migration of leukocytes by binding to VE-cadherin in vascular endothelial cells (60). Additionally, Fg activates AKT signaling and induces microfilament depolymerization, thereby enhancing endothelial barrier permeability (78).

**Figure 1:**
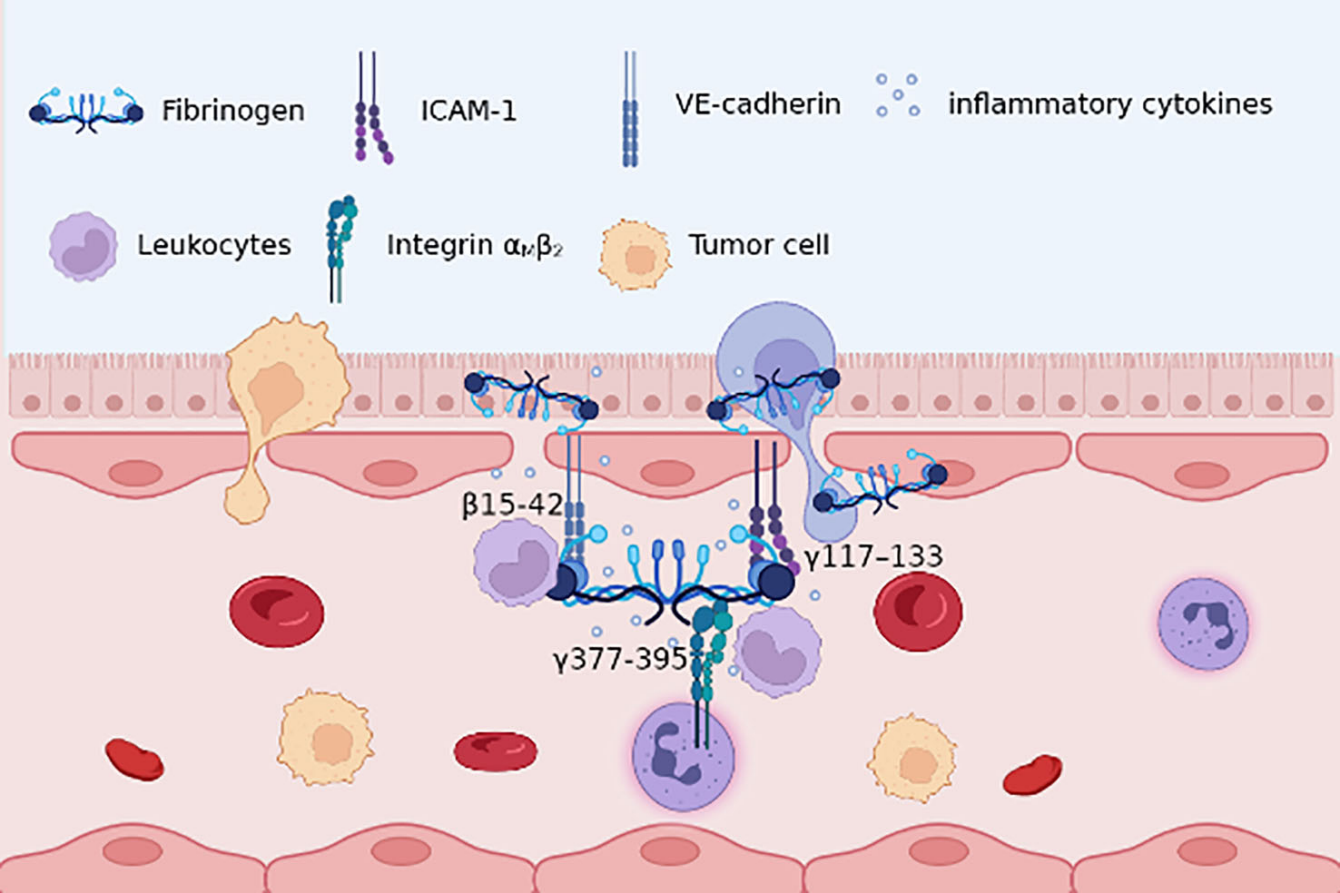
Fg β15-42, γ117–133 with endothelial cells VE-cadherin, ICAM-1, respectively, improves endothelial barrier permeability; γ377-395 with leukocytes integrin αMβ2 promotes adhesion and migration. Besides, Fg may lead to the recruitment of pro-inflammatory cytokines, thus providing an inflammatory role in TME.

Recent evidence has highlighted the association between circulating Fg and DNA methylation in peripheral blood leukocytes (79), underscoring Fg’s role in leukocyte migration and recruitment. Acting as a bridging molecule, Fg links leukocyte integrin αMβ2 (Mac-1) to intercellular adhesion molecule-1 (ICAM-1) on endothelial cells (ECs) (80), facilitating leukocyte adhesion and transvascular migration across the vascular endothelium. Moreover, at sites of inflammation, Fg interacts with immune cells, hastening their recruitment to participate in the inflammatory cascade (81). (**Figure 1**) A reciprocal relationship exists between leukocytes and Fg, wherein during inflammation, reactive oxygen species (ROS) from neutrophils and monocytes, along with nitric oxide from lymphocytes and monocytes, cleave Fg (82). Subsequently, protease-cleaved Fg binds to macrophage toll-like receptors, amplifying the inflammatory response through an allergic cascade (83). This establishes a positive feedback loop between Fg and immune cells, intensifying the inflammatory milieu.

Moreover, inflammatory mediators play crucial regulatory roles in both vascular and leukocyte responses. Fg has been implicated in promoting inflammatory responses by enhancing the secretion of inflammatory factors, either independently or in response to various environmental cues.

Studies have demonstrated that Fg upregulates the secretion of several cytokines, including IL-6, IL-17, IL-23, TNF-α, macrophage inflammatory protein-1 α (MIP-1α), MIP-1β, MIP-2, and monocyte chemotactic protein-1 (MCP-1) (84–86). The molecular mechanisms underlying Fg-mediated promotion of inflammatory factors have also been elucidated. Fg participates in the inflammatory process by activating the NF-κB pathway and inducing the expression of interleukin-6 (IL-6), interleukin-8, monocyte chemotactic protein-1 (MCP-1), and C-C chemokine ligand-2 (CCL2) (87, 88).

Furthermore, Fg has been found to stimulate cytokine secretion under various conditions. Specific chemical modifications, such as D-ibose glycosylation, can enhance Fg’s involvement in specific inflammatory responses, resulting in increased expression of inflammation-associated cytokine genes, including TNF-α, IL-6, IL-1β, and IFN-γ (89). Additionally, specific enzymatic structures, such as atypical cross-linking mediated by the glutamine transferase transglutaminase-2 (TG2), enhance the secretion of pro-inflammatory cytokines (e.g. TNF-α) in myeloid macrophages, while simultaneously reducing the expression of IL-10 and inhibiting the phosphorylation of STAT3 (90).

In the specific context of a tumor, the role of Fg and immune cells undergo alterations. Typically, during inflammation, Fg activates macrophages, polarizing them into pro-inflammatory M1 macrophages via integrin α1β3. This activation leads to the expression of M1 macrophage-specific markers such as inducible nitric oxide synthase (iNOS) and pro-inflammatory cytokines (IL-1β, IL-6, TNF-α) (91). However, within the tumor microenvironment, Fg becomes associated with increased infiltration of tumor-associated macrophages (TAMs) exhibiting an M2 phenotype. These M2 TAMs promote tumor growth, induce angiogenesis, and suppress immune responses (20).

Importantly, within the specific context of a tumor, the interplay between Fg and immune cells may contribute to tumor development. Distinct interactions occur between Fg γ^390-396A^ and leukocyte integrin αMβ2 (Mac-1) (92), typically mediated by Fg binding to CD11b (93). These interactions lead to the activation of downstream proteins, including focal adhesion kinase (FAK) phosphorylation and mitogen-activated protein kinase (MAPK) activation. Consequently, this triggers various processes such as leukocyte degranulation, recruitment (94), phagocytosis, and induction of inflammatory responses (95). Specifically, within the tumor microenvironment, Fg γ^390-396A^’s interaction with leukocyte integrin αMβ2 not only enhances the secretion of pro-inflammatory cytokines like IL-6, IL-1β, IFN-γ, and TNF-α but also promotes tumor cell proliferation (96). This dual role underscores Fg’s contribution to inflammation and tumor development within tumors.

### 2.4 Fg and angiogenesis

The relationship between Fibrinogen (Fg) and angiogenesis remains a subject of ongoing debate, with recent research shedding light on its dual role—both pro- and anti-angiogenic—in specific tissue contexts, particularly within the spectrum of various diseases.

The role of Fg in angiogenesis varies depending on the environmental context. In skin wound models, a distinctive pattern emerges as Fg infiltrates the lesion during the inflammatory phase, coinciding with a decline in vascular integrity. Notably, this Fg deposition correlates with heightened re-epithelialization and accelerated angiogenesis, emphasizing its context-dependent pro-angiogenic effects (97). Contrasting observations arise in a colon cancer MC38 model, where mice deficient in Fg exhibit lower vascular density compared to control mice. This effect is found to be time-dependent (98), further illustrating the intricate dynamics involved.

Fg demonstrates a pro-angiogenic effect under certain conditions. Recent insights highlight the direct interaction of circulating Fg with various growth factors, encompassing members of the vascular endothelial growth factor (VEGF) family and fibroblast growth factor (FGF). This interaction stimulates endothelial cell proliferation, fostering angiogenesis, and fueling tumor cell growth (99, 100). Mechanistically, tumor-derived Fg amplifies the impact of FGF-2 on endothelial proliferation through coordinated effects involving integrin αvβ3 and FGFR1 (FGF receptor 1) (101).

Moreover, Fg can promote angiogenesis through alternative mechanisms. Fg collaborates with immune cells to orchestrate a dual-promotional role in immune cell recruitment and angiogenesis (102). This multifaceted engagement includes the inhibition of tumor angiogenesis inhibitors, such as Endostatin, by Fg, thereby promoting angiogenic aggregation (103).

Additionally, specific fragments of Fg have been implicated in promoting angiogenesis. Fg α component enhances mesenchymal cell-endothelial cell interactions, actively participating in angiogenesis through the VEGA-VEGFR-FAK signaling axis (104).Exploring the intricate landscape of Fg binding sites involved in tumor angiogenesis reveals a nuanced interplay of molecular interactions that influence endothelial cell behavior. One reported interaction spotlights arginine-glycine-aspartic acid (RGD) site in at α252-254 or α572–574 region, showcasing its direct engagement with integrin αvβ3. This interaction holds the potential to function as a survival signal for endothelial cells, orchestrating pivotal roles in the angiogenic process (105).

**Figure 2:**
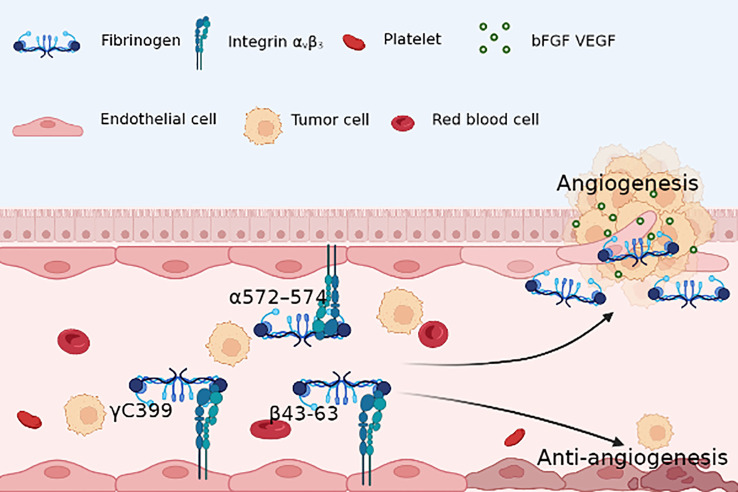
Fg RGD sequence(α572–574) with endothelial cells integrin αvβ3 provides survival signal for ECs and activates microvascular ECs, contributing to angiogenesis; however, Fg γC399 and β43-63 induce apoptosis of ECs, which results in an anti-angiogenesis environment.

Contrastingly, some studies challenge the notion of Fg’s indispensability for angiogenesis, suggesting potential anti-angiogenic roles. Integrin αvβ3 serves as a pivotal receptor that mediates the interaction between Fg and angiogenesis, with different fragments of Fg exhibiting distinct effects upon binding to this receptor. Interestingly, while certain Fg fragments have been associated with pro-angiogenic effects through integrin αvβ3 binding, others have been linked to the inhibition of tumor angiogenesis. An intriguing find lies in the β43-63 region, identified as a novel anti-tumor peptide due to its anti-angiogenic activity mediated by integrin αvβ3 (106). Additionally, the γC component and its truncation mutant (γC399tr) emerge as determinants in the binding of Fg to endothelial cells via integrin αvβ3. This interaction, in turn, contributes to endothelial cell apoptosis and a reduction in tube formation (107) (**Figure 2**).

Moreover, certain fragments of Fg have been reported to be linked to the inhibition of tumor angiogenesis. A key functional fragment, Fg α1-24, known as alphastatin, exhibits inhibitory effects on bFGF- and VEGF-induced migration, proliferation, and tubule formation of microvascular endothelial cells (108). This inhibition is mediated through the suppression of the JNK and ERK kinase pathways (109). Alphastatin’s localized effects extend to cellular necrosis, thrombosis, and vessel rupture (110), collectively acting as a potent inhibitor of angiogenesis and tumor growth (111). Notably, the down-regulation of Fg E-fragment in tumors (112) hinted at its detrimental impact on tumor growth. Subsequent investigations propose that the Fg E fragment might be linked to the inhibition of tumor angiogenesis.

Despite numerous studies reporting either promotion or inhibition of angiogenesis by Fg and its fragments, there are also studies indicating that Fg may not be significantly associated with angiogenesis in certain contexts. Tumor analysis from Fg-deficient mice and normal controls exhibited no genotype-dependent variations in vessel density and pattern (113). In regions with heightened Fg leakage, quantifying vascular density revealed no alterations in blood vessel size or density, dissociating elevated plasma Fg from angiogenesis (114).

### 2.5 Fg and metastasis

The coagulation cascade and platelet activation stand as pivotal drivers of metastasis, orchestrating a multifaceted interplay in tumor progression. Fibrinogen(Fg), a crucial constituent of the tumor microenvironment, facilitates tumor metastasis via both platelet-dependent and platelet-independent pathways.

Initially, Fg may foster tumor metastasis through platelet-dependent mechanisms. Platelets, circulating in the bloodstream, emerge as essential accomplices in facilitating tumor metastasis, underscoring their intricate involvement in the metastatic cascade. Platelet membranes host a myriad of adhesion molecules, including integrin α_6_β_1_, α_v_β_3_and α_IIb_β_3_, emerges as a critical mediator in platelet-tumor cell interactions, particularly through its facilitation of integrin α_v_β_3_ binding to tumor cells (115). Moreover, he interaction between platelet integrin α_IIb_β_3_ and specific Fg sequences, such as γA400-411 and arginine-glycine-aspartic acid(RGD) (116), enhances cancer cell adhesion to endothelial cells, fostering the dissemination of tumor cells (116, 117). Notably, studies in mice lacking Fg and Gαq, a G protein pivotal for platelet activation, showcased significantly reduced tumor cell survival compared to control counterparts (118, 119). This underscores the potential of Fg in bolstering platelet-tumor cell interactions, thereby modulating the metastatic potential mediated by platelets.

**Figure 3:**
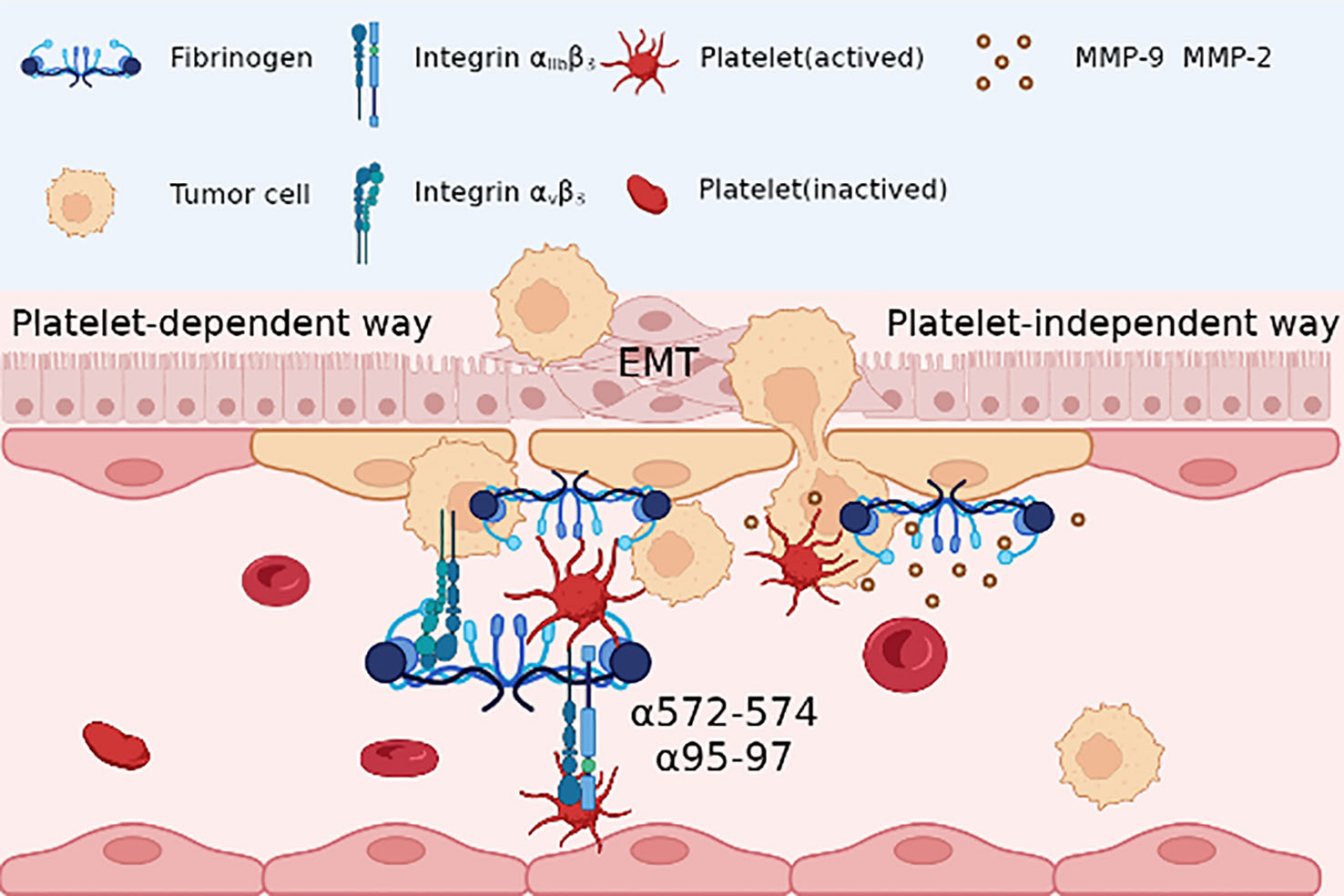
In platelet dependent way, Fg acts as a bridge between the reaction between tumor cells and platelet, with adhesion to tumor integrin αvβ3 and RGD sequence(α572-574, α95-97) binding to platelet integrin αIIbβ3; in platelet-independent way, Fg may be together with matrix metalloproteinase (MMP)-2 and MMP-9, promoting the epithelial-mesenchymal transition (EMT) and migration of tumor cells.

However, divergent perspectives exist regarding the role of Fg in cancer metastasis independent of platelets. Fg β15-42 emerges as a key player in augmenting vascular permeability and facilitating tumor cell migration across endothelial cells by binding to VE-cadherin (60). Furthermore, Fg serves as a mediator for matrix metalloproteinase (MMP)-2 and MMP-9, pivotal enzymes involved in tumor metastasis, by participating in extracellular matrix remodeling (74). These findings underscore Fg’s intricate involvement in regulating tumor cell migration during metastasis by integrating into the ECM and modulating matrix composition (120) (**Figure 3**).

Furthermore, Fg can directly interact with tumor cells, thereby enhancing their metastatic potential. Fg plays a pivotal role in fostering persistent adherence, survival of metastatic emboli post-tumor cell intravasation, and contributes significantly to spontaneous metastasis (25, 27). Interactions between cancer cells and Fg, particularly in cases where cancer cells express ICAM-1, expedite endothelial penetration for metastasis by forming Fg-dependent bridges (121). Furthermore, the citrullination of Fg by lung endothelial cells promotes tumor cell aggregation and enhances metastatic potential (122).

Insights derived from studies involving Fg-deficient mice provide valuable illumination into the multifaceted role of Fg in metastasis. These investigations have yielded evidence showcasing a measurable reduction in lung micro-metastasis or overall tumor burden in the absence of Fg (113, 123). Crucially, this quantitative decline doesn’t correlate with observable changes in tumor stroma development or the general growth pattern of tumors. It’s noteworthy that the reduction in metastatic occurrences, though significant, falls short of achieving complete elimination. This intriguing observation was consistent across models involving Lewis lung carcinoma and the B16-BL6 melanoma, indicating a nuanced relationship between Fg and the metastatic process (124). This nuanced interplay suggests that while Fg deficiency exerts a discernible impact, it does not entirely eradicate metastatic events, prompting a deeper exploration into the specific mechanisms and contexts governing the complex interaction between Fg and metastasis.

Contrastingly, evidence supporting an anti-metastatic role for Fg and its fragments also exists. In a Met-1 breast cancer nude mouse model injected with the γC truncation mutant (γC399tr), reduced metastasis was observed compared to control mice, indicating the potential of γC399tr in impeding tumor growth (107). Additional findings revealed that *in vivo* subcutaneous injection of fibrinogen Aα(FGA) knockout (KO) A549 cells into immunodeficient BALB/c nude mice led to increased A549 cell proliferation and colonization, suggesting an inhibitory role of Fg in this context (125). Moreover, a unique perspective posits Fg’s anti-metastatic influence in anatomical sites characterized by low Fg concentrations, such as lymphatics and body cavities. Schneider et al.’s hypothesis suggesting the suppression of tumor cell migration by Fg, acting as a counterforce to free vitronectin, adds a layer of complexity to our understanding of Fg’s diverse roles in metastasis (126).

These multifaceted observations underscore the intricate and context-dependent nature of Fg’s impact on metastatic processes, warranting further exploration to decipher the nuanced interplay between Fg and tumor metastasis.

### 2.6 Molecular pathways of Fg in regulation of tumor progression

Currently, investigations into the relationship between Fibrinogen (Fg) and tumors predominantly center around clinical data analysis, with a limited number of studies delving into the intricate molecular mechanisms at play. In diverse tumor microenvironments, the molecular pathways involving Fg exhibit variability, indicating the multifaceted role of Fg in tumorigenesis.

In the context of lung adenocarcinoma (LUAD), Fg typically promotes tumor proliferation and metastasis, Fibrinogen Aα(FGA) exerts inhibitory effects on tumor growth, metastasis, and invasion while fostering apoptosis through the suppression of the PI3K-AKT-mTOR pathway (125).

Shifting focus to esophageal squamous cell carcinoma (ESCC), Fg demonstrates a distinctive impact. Here, Fg is implicated in the promotion of cell migration and invasion, albeit without a significant influence on proliferation. This nuanced effect is achieved through the induction of Epithelial-Mesenchymal Transition (EMT) via the p-AKT/p-mTOR pathway (8). In some ESCC patients with elevated Fg, TP53, KMT2D, and NOTCH1 actionable gene variants were detected, but the specific signaling pathways remain to be studied (20).

Moving to colon cancer, the interaction between Fg and tumor cells is facilitated by the activation of Focal Adhesion Kinase (FAK), concurrently inhibiting the tumor suppressor p53 and its downstream targets, such as 14-3-3σ and p21. This concerted action promotes unbridled cell growth while inhibiting senescence (67).

Within the intricacies of the hepatocellular carcinoma (HCC) microenvironment, Fg emerges as a pivotal player, exerting its influence through interactions with integrin αvβ5 and subsequent activation of hepatic stellate cells (HSCs). This interaction establishes a robust correlation with both the incidence and recurrence of HCC in affected individuals (5). Fg’s impact extends beyond mere association, actively promoting migration and invasion of hepatocellular carcinoma cells. These pro-metastatic effects are mediated through the activation of the PTEN/AKT/mTOR pathway, coupled with the induction of epithelial-mesenchymal transition (EMT) (127). Furthermore, in hepatoma cell lines, the expression of Fg undergoes significant modulation. This modulation is intricately linked to the degradation of signal transducer and activator of transcription (STAT), providing a glimpse into the regulatory mechanisms governing Fg expression (128). Significantly, the targeted inhibition of p38 MAPK demonstrates a marked ability to downregulate Fg expression, thereby illuminating a promising avenue for precision therapeutic interventions (128). This finding underscores the potential interplay between Fg and key signaling pathways, including JAK/STAT and p38 MAPK, hinting at intricate regulatory mechanisms within the cellular milieu. In addition, in HCC, fibrinogen Aα(FGA) can act as a tumor suppressor with opposite effects. It has been shown that FGA mutations can promote hepatocellular carcinoma development by activating TYK2-STAT3 signaling and increasing IL-6 expression (129). Additionally, FGA can impede AKT phosphorylation and its downstream effectors, such as Bcl-2, by binding to HBsAg, thereby diminishing pro-survival protein levels while augmenting pro-apoptotic protein expression (130). The intricate interplay of these molecular events highlights Fg’s multifaceted role in shaping the aggressive behavior of hepatocellular carcinoma.

Within gallbladder cancer (GBC) cells, Fg orchestrates a multifaceted interplay, aggregating macrophages to propel angiogenesis. This effect is mediated through the upregulation of Intercellular Adhesion Molecule-1 (ICAM-1) expression (131). Moreover, Fg induces a phenotypic shift in GBC cells, typified by an increase in the mesenchymal marker vimentin, coupled with a concomitant decrease in the epithelial marker E-cadherin. This dual modulation strongly implies that Fg plays a contributory role in cell migration and invasion, potentially through the induction of Epithelial-Mesenchymal Transition (EMT) signaling (132).

Within the intricate milieu of ovarian cancer, Fg emerges as a dynamic orchestrator, stimulating fibroblasts to enhance the production of Type I alpha 1 collagen (COL1A1). Simultaneously, Fg acts as a catalyst, activating the AKT signaling pathway (133). These concerted actions contribute to the facilitation of cancer metastasis, marking Fg as a key player in shaping the metastatic landscape of ovarian cancer.

In breast cancer, the extracellular matrix, including Fg, plays a pivotal role in modulating tumor cell behavior, particularly proliferation and quiescence, through mechanical stimuli. Lower mechanical forces stimulate the cytoskeleton/AIRE axis via integrin β1/3 receptor activation, enhancing tumor cell potential. Conversely, excessive mechanical forces activate DDR2/STAT1/P27 signaling, inducing cell cycle arrest and transitioning stem cell-like cancer cells into a quiescent state (134).

**Figure 4:**
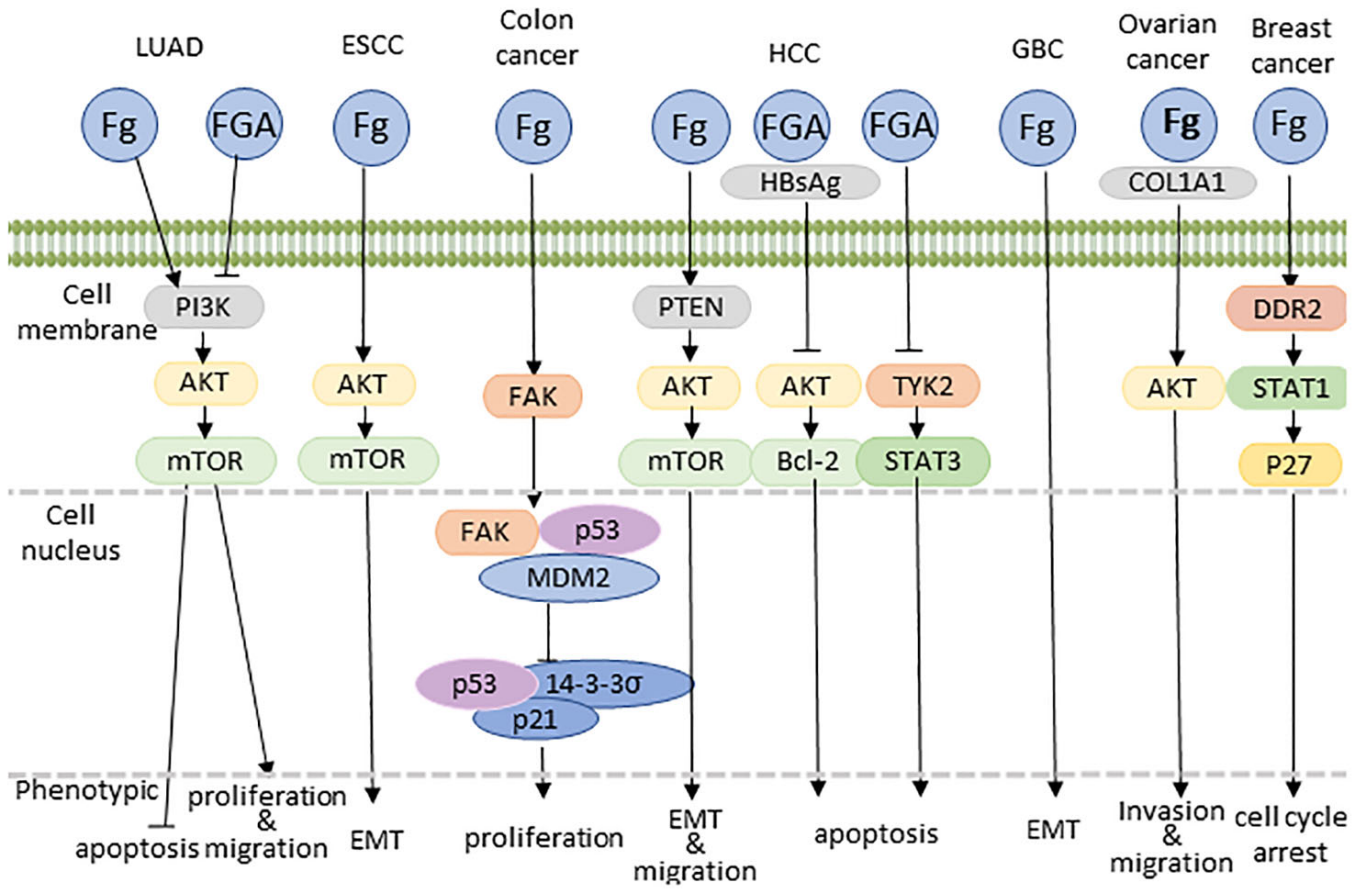
Proposed model explaining Fg-driven tumor growth. COL1A1, type I alpha 1 collagen; EMT, epithelial to mesenchymal transition.

This nuanced understanding of Fg regulation offers valuable insights into the potential synergies between different signaling cascades (**Figure 4**), providing a foundation for the development of targeted therapeutic strategies aimed at modulating Fg-mediated pathways in disease states.

## 3 Discussion

The existing comprehension of the relationships between Fibrinogen (Fg) and tumors primarily rests on clinical data, consistently indicating that elevated plasma Fg levels serve as independent predictors for an unfavorable prognosis across various tumor types. A mounting body of evidence underscores Fg’s multifaceted role in promoting tumorigenesis, encompassing pro-inflammatory, metastatic, and angiogenic effects that collectively contribute to tumor progression.

Based on our thorough literature review, we anticipate significant potential for further development in understanding the relationship between Fg and tumors. Primarily, in clinical contexts, Fg determination methods need not be confined solely to plasma analysis, such as the traditional Clauss method; exploring liquid biopsy (135) or salivary Fg detection (136) could shed light on Fg’s prognostic implications for tumors. There is a pressing need for more comprehensive studies on how Fg levels may interfere with various chemotherapy regimens and impact the development of therapy resistance. Furthermore, future investigations could delve into the prognostic significance of plasma Fg ratios when combined with other tumor prognostic markers, such as the fibrinogen-to-albumin ratio (137, 138), F-NLR (fibrinogen and neutrophil-lymphocyte ratio) (139), among others. Additionally, there is scope for further exploration of the roles played by Fg fragments, including FGA, FGB, and FGG, in the context of tumor prognosis.

In terms of basic research, while the prognostic significance of plasma Fg in various tumors has been extensively investigated, several areas warrant further exploration. These include elucidating the mechanisms underlying the association between elevated Fg levels prior to treatment and the subsequent poor prognosis of cancer patients post-surgery or chemotherapy. Additionally, the mechanisms governing Fg deposition within the microenvironment of diverse tumor types, as well as its involvement in processes such as inflammation, angiogenesis, and metastasis, require thorough investigation. Future studies could focus on uncovering the intricate molecular mechanisms through which Fg influences tumor progression across different cancer types. This could involve identifying specific integrins that interact with Fg, elucidating downstream effector molecules modulated by Fg, and assessing the potential relevance of these Fg-mediated signaling pathways in various human tumors. Furthermore, in terms of experimental protocol development, research targeting Fg as a therapeutic target has primarily been limited to a few tumor cell lines or murine models, neglecting the broader spectrum of tumor types. Given the challenges associated with Fg deficiency and its implications for hemostasis, refining experimental protocols is imperative. More precise methodologies are urgently needed to facilitate *in situ* injection of cancer cell lines into Fg-knockout mice, enabling a more comprehensive understanding of Fg’s role in tumor biology. Lastly, there is scope to expand investigations into the interactions between Fg-related fragments and tumors, which may yield novel insights. Exploring these interactions could uncover unexpected findings and contribute to a deeper understanding of the complex interplay between Fg and tumorigenesis.

The translation of relevant research findings on Fg and tumors into clinical applications is a promising endeavor that can significantly impact tumor treatment protocols. Firstly, our review findings suggest a strong association between elevated pretreatment Fg levels and poor prognosis in most tumor patients. This association can serve as a valuable guide for determining treatment regimens and post-treatment management strategies for cancer patients. Additionally, compared to current tumor markers that often involve cumbersome testing steps like immunohistochemistry, assessing Fg levels through pre-treatment coagulation is more convenient, accessible, and provides a quicker reference value. Secondly, Fg can serve as a tool for stratifying and assessing individual tumor risk. Particularly in cases where tumors lack precise and specific prognostic biomarkers, plasma Fg offers significant clinical utility. Furthermore, specific fragments of Fg, such as FGA, FGB, and FGG, may exert distinct effects on tumors compared to intact Fg. For instance, FGA may function as an inhibitory factor, presenting itself as a potential target for clinical interventions and tumor treatments. Lastly, targeting Fg to mitigate its role in promoting tumorigenesis and development holds promise for innovative cancer treatments. Strategies like partial inhibition of the Fg gene or blocking downstream signaling pathways activated by Fg could prevent its involvement in tumor metastasis and angiogenesis. These approaches offer new avenues for cancer treatment and have the potential to enhance the effectiveness and customization of therapeutic strategies.

## Author contributions

XW: Writing – review & editing, Writing – original draft. XY: Writing – review & editing, Writing – original draft. CC: Writing – review & editing, Writing – original draft. CIC: Writing – review & editing, Writing – original draft. YW: Writing – review & editing, Writing – original draft. DS: Writing – review & editing, Writing – original draft. LZ: Writing – review & editing, Writing – original draft.
